# Association of *DIAPH1* gene polymorphisms with ischemic stroke

**DOI:** 10.18632/aging.102631

**Published:** 2020-01-03

**Authors:** Zhanyun Ren, Xiaotian Chen, Wuzhuang Tang, Jie Li, Song Yang, Yanchun Chen, Xianghai Zhao, Huihua Zong, Chunlan Liu, Chong Shen

**Affiliations:** 1 Department of Neurology, Affiliated Yixing Peopleʼs Hospital of Jiangsu University, Peopleʼs Hospital of Yixing City, Yixing 214200, China; 2 Department of Clinical Epidemiology, Childrenʼs Hospital of Fudan University, Shanghai 201102, China; 3 Department of Epidemiology, School of Public Health, Nanjing Medical University, Nanjing 211166, China; 4 Department of Cardiology, Affiliated Yixing Peopleʼs Hospital of Jiangsu University, Peopleʼs Hospital of Yixing City, Yixing 214200, China

**Keywords:** *DIAPH1*, polymorphisms, stroke, mRNA

## Abstract

DIAPH1 is a formin protein involved in actin polymerization with important roles in vascular remodeling and thrombosis. To investigate potential associations of *DIAPH1* single-nucleotide polymorphisms (SNPs) with hypertension and stroke, 2,012 patients with hypertension and 2,210 controls, 2,966 stroke cases [2,212 ischemic stroke (IS), 754 hemorrhagic stroke (HS)] and 2,590 controls were enrolled respectively in the case-control study. A total of 4,098 individual were included in the cohort study. *DIAPH1* mRNA expression was compared between 66 IS [43 small artery occlusion (SAO) and 23 large-artery atherosclerosis (LAA)] and 58 controls. Odds ratio (*OR*), hazard ratio (*HR*) and 95% confidence interval (*CI*) were calculated by logistic and cox regression analysis. Rs7703688 T>C variation was significantly associated with an increased risk of IS [*OR* (95% *CI*) was 1.721 (1.486-1.993), *P*=4.139×10^-12^]. Association of rs7703688 with stroke risk was further validated in the cohort study [adjusted *HRs* (95% *CI*s) for additive and recessive models were 1.385 (1.001-1.918), *P*=0.049, and 2.882 (1.038-8.004), *P*=0.042, respectively)]. *DIAPH1* mRNA expression was significantly downregulated in IS. In SAO stroke subtype, *DIAPH1* expression has an increased trend among rs251019 genotypes (*P_trend_*=0.048). These novel findings suggest that *DIAPH1* variation contributes to genetic susceptibility to stroke risk, especially the SAO subtype of IS.

## INTRODUCTION

Stroke ranks as the first leading cause of death in China, with bearing the highest stroke burden in the world [1]. Hypertension is a major risk factor for cerebrovascular disease, including ischemic stroke (IS) and hemorrhagic stroke (HS) [2]. However, characterization of the mechanisms underlying stroke is still incomplete.

Chronic hypertension induces vascular remodeling of cerebral arteries, an essential risk factor for stroke. At play in this phenomenon is activation of the Rho/Rho-kinase pathway, a crucial modulator of proliferation, motility, and contractility of smooth muscle cells (SMCs) [3–5]. Supporting a key association between Rho/Rho-kinase pathway and cardiovascular diseases via vascular remodeling, our previous studies showed that genetic variations in Rho kinases (*ROCK1* and *ROCK2*) modulate susceptibility to hypertension and stroke [6].

Mammalian homolog of Drosophila diaphanous 1 (DIAPH1), a formin protein, is a canonical effector for Rho signaling in humans [7, 8]. After being activated by GTP-bound RhoA, the formin homology-2 domain of DIAPH1 stimulates actin filament assembly at the barbed ends [9]. DIAPH1 mediates vascular remodeling via integration of oxidative stress and signal transduction pathways in SMCs [10]. Besides, DIAPH1 induces pro-platelet formation in megakaryocytes by coordinating the actin and microtubule cytoskeleton [11], which critically impacts blood clotting and thrombogenic processes.

Previous studies have focused on the association of *DIAPH1* polymorphisms and macrothrombocytopenia, hearing loss, blindness, and cancer [12–14]. Importantly, animal experiments have demonstrated that genetic deletion of *DIAPH1* led to infarct size reduction and improved contractile function after myocardial ischemia/reperfusion [15]. However, whether changes in *DIAPH1* expression or function may contribute to stroke incidence has not been established. On account of the important role of *DIAPH1* on vascular remodeling and thrombosis, i.e. two key aspects in the pathophysiology of stroke, we decided to investigate potential associations between *DIAPH1* gene variations and stroke risk.

To this end, we performed case-control and cohort studies to evaluate the association of single-nucleotide polymorphisms (SNPs) in the human *DIAPH1* gene with susceptibility to hypertension and stroke. In addition, the distribution of *DIAPH1* SNP genotypes was typified by measuring *DIAPH1* mRNA expression in peripheral blood mononuclear cells (PBMCs) from IS and hypertensive controls. The present findings provide novel insights about the potential contribution of *DIAPH1* polymorphisms to the pathogenesis of hypertension and stroke.

## RESULTS

### Demographic and clinical characteristics of the study population

Clinic-demographic characteristics of participants in the hypertension case-control study are summarized in Supplementary Table 1. Although study subjects were matched for age (5 year-group), hypertensive cases were on average 3.42 years older than controls (*P* < 0.001). Participants with hypertension had higher BMI, total cholesterol (TC), triglycerides (TG), low-density lipoprotein cholesterol (LDL-C), glucose (GLU), and a higher rate of type 2 diabetes mellitus (T2DM) than controls (*P* < 0.001). No significant differences in gender, high-density lipoprotein cholesterol (HDL-C), or smoking and drinking statuses were observed (*P* > 0.05).

The characteristics of individuals in the stroke case-control study are summarized in Supplementary Table 2. Significant differences were observed among IS, HS, and controls for age, gender, smoking and drinking habits, hypertension, lipid profiles, and T2DM (*P* < 0.05). Post-hoc multiple comparisons showed that total TC, HDL-C, and LDL-C levels were significantly higher in IS cases than in controls. Compared to controls, HS cases were older, had higher levels of TC and HDL-C, and lower TG levels. These characteristics were adjusted as confounding factors when evaluating the association of *DIAPH1* with stroke.

### Association analysis of *DIAPH1* variants in the case-control study of hypertension

In the case-control study of hypertension, the allele frequencies of the *DIAPH1* rs3805691, rs251019, and rs11954998 SNPs in controls were consistent with Hardy-Weinberg equilibrium (*HWE*), whereas the allele frequencies of rs251018 and rs7703688 were not (*HWE* *P* = 0.004 and *P* < 0.001, respectively). Compared with CC/CT carriers, the rs251019 TT genotype was significantly associated with decreased risk of hypertension, after adjusting for covariates including age, gender, BMI, TC, TG, HDL-C, LDL-C, GLU, smoking status, and drinking status [adjusted odds ratio (*OR*), 0.791; 95% confidence interval (*CI*), 0.636-0.984; *P*=0.035] (Table 1).

**Table 1:** Association analyses of *DIAPH1* SNPs with hypertension in the case-control study.

**SNP**	**Group**	**WT/HT/MT**	***OR*(95% *CI*)^a^**			
			**Additive model**	**Dominant model**	**Recessive model**	***P*-values^b^**
rs3805691		CC/CT/TT				
	Case	1136/749/126	1.000(0.902-1.109)	0.994(0.875-1.13)	1.031(0.793-1.341)	0.805
	Control	1253/825/132	*P*=0.996	*P*=0.931	*P*=0.820	
rs251018		TT/TG/GG				
	Case	1421/551/40	0.953(0.845-1.075)	1.001(0.878-1.158)	0.572(0.384-0.812)	0.004
	Control	1556/574/79	*P*=0.435	*P*=0.906	*P*=0.006	
rs251019		CC/CT/TT				
	Case	980/866/166	0.946(0.859-1.042)	0.985(0.868-1.117)	0.791(0.636-0.984)	0.249
	Control	1055/927/228	*P*=0.261	*P*=0.813	*P*=0.035	
rs7703688		TT/TC/CC				
	Case	1672/330/10	0.893(0.766-1.040)	0.942(0.797-1.113)	0.315(0.152-0.650)	<0.001
	Control	1818/356/35	*P*=0.146	*P*=0.485	*P*=0.002	
rs11954998		TT/TC/CC				
	Case	1482/488/42	0.943(0.830-1.071)	0.920(0.798-1.060)	1.112(0.710-1.742)	0.205
	Control	1590/578/42	*P*=0.366	*P*=0.248	*P*=0.642	

WT wild type, HT heterozygote, MT mutant type;

Rs251018 and rs7703688 were not consistent with *HWE,* with *P* values of 0.004 and 0.001.

a: Adjusted for age, gender, BMI, GLU, HDL-C, LDL-C, TC, TG, smoking status and drinking status.

b: The *P*-values of *HWE* test in controls.

### Association analysis of *DIAPH1* variants in the case-control study of IS

In the case-control study of stroke, the frequencies of all *DIAPH1* SNPs in controls were consistent with *HWE*. Table 2 shows the results of the association analyses after adjusting for age, gender, smoking status, drinking status, TC, TG, LDL-C, HDL-C, T2DM, and hypertension. The additive model (CC *vs* CT *vs* TT) suggested that the rs3805691 variant was associated with decreased risk of IS (adjusted *OR=*0.782, 95% CI=0.700–0.874, *P=*1.3×10^-5^). Compared with CC carriers, CT/TT carriers of the rs3805691 SNP were also at lower risk of IS (adjusted *OR*=0.712, 95% *CI*=0.622–0.815, *P*=7.986×10^-7^). Comparable protective effects against stroke were observed for the rs251019 variant in both additive and dominant models. In contrast, rs7703688 and rs11954998 C allele carriers were associated with increased risk of IS [*ORs* (95% *CI*s) for the additive model were 1.721 (1.486-1.993) and 1.537 (1.356-1.743), *P*=4.139×10^-12^ and *P*=2.058×10^-11^, respectively]. These associations were still significant after Bonferroni correction. No significant association with IS was found for rs251018, nor between the five *DIAPH1* SNPs and HS.

**Table 2:** Association analyses of *DIAPH1* SNPs with stroke sub-types in the case-control study.

**Stroke subtypes**	**SNP**	**Group**	**WT/HT/MT**	***OR* (95% *CI*)^a^**		
				**Additive model**	**Dominant model**	**Recessive model**
IS	rs3805691		CC/CT/TT			
		Case	1429/623/109	0.782(0.700-0.874)	0.712(0.622-0.815)	0.883(0.666-1.171)
		Control	1434/990/166	*P*=1.300×10^-5^	*P*=7.986×10^-7^	*P*=0.388
	rs251018		TT/TG/GG			
		Case	1533/619/46	1.041(0.916-1.182)	1.066(0.923-1.232)	0.893(0.579-1.378)
		Control	1857/663/70	*P*=0.539	*P*=0.386	*P*=0.609
	rs251019		CC/CT/TT			
		Case	1322/681/206	0.813(0.734-0.900)	0.685(0.600-0.782)	1.084(0.862-1.362)
		Control	1277/1082/231	*P*=6.200×10^-5^	*P*=1.899×10^-8^	*P*=0.491
	rs7703688		TT/TC/CC			
		Case	1638/509/61	1.721(1.486-1.993)	1.771(1.504-2.086)	3.076(1.790-5.287)
		Control	2164/401/25	*P*=4.139×10^-12^	*P*=6.923×10^-12^	*P*=4.800×10^-5^
	rs11954998		TT/TC/CC			
		Case	1336/763/60	1.537(1.356-1.743)	1.626(1.410-1.874)	1.752(1.159-2.650)
		Control	1893/644/53	*P*=2.058×10^-11^	*P*=2.275×10^-11^	*P*=0.008
HS	rs3805691		CC/CT/TT			
		Case	411/296/41	0.979(0.809-1.185)	1.023(0.808-1.295)	0.771(0.449-1.322)
		Control	1434/990/166	*P*=0.830	*P*=0.850	*P*=0.344
	rs251018		TT/TG/GG			
		Case	548/176/24	0.941(0.745-1.189)	0.945(0.726-1.231)	0.831(0.372-1.855)
		Control	1857/663/70	*P*=0.978	*P*=0.677	*P*=0.652
	rs251019		CC/CT/TT			
		Case	392/284/67	0.972(0.814-1.160)	0.940(0.743-1.188)	1.042(0.691-1.569)
		Control	1277/1082/231	*P*=0.754	*P*=0.604	*P*=0.845
	rs7703688		TT/TC/CC			
		Case	610/126/15	1.103(0.832-1.463)	1.078(0.787-1.476)	1.633(0.605-4.411)
		Control	2164/401/25	*P*=0.494	*P*=0.640	*P*=0.333
	rs11954998		TT/TC/CC			
		Case	547/181/20	1.101(0.870-1.393)	1.107(0.853-1.438)	1.238(0.549-2.791)
		Control	1893/644/53	*P*=0.424	*P*=0.445	*P*=0.606

IS: ischemic stroke; HS: hemorrhagic stroke. The allele frequencies of all SNPs in controls were consistent with *HWE*; *HWE P*-values for rs3805691, rs251018, rs251019, rs7703688, and rs11954998 were 0.780, 0.244, 0.933, 0.184, and 0.837 respectively.

a: Adjusted for age, gender, smoking status, drinking status, TC, TG, LDL-C, HDL-C, T2DM, and hypertension.

Furthermore, we conducted SNP association analysis stratified by TOAST subtypes (Supplementary Table 8). After covariates adjustment, both rs3805691 and rs251019 were negatively associated with small artery occlusion (SAO) and large-artery atherosclerosis (LAA) under the additive and dominant models. Meanwhile, the rs11954998 and rs7703688 variants were instead associated with increased risks of SAO and LAA under all three genetic models, while no association was detected between rs251018 and either SAO or LAA. Analysis for HS subtypes showed that compared to the TT/TC genotypes, the CC genotype of rs7703688 conferred higher risk for subarachnoid hemorrhage (SAH), whereas none of the *DIAPH1* SNPs studied showed association with intracerebral hemorrhage (ICH) (Supplementary Table 9).

### Association analysis of *DIAPH1* variants in the cohort study of hypertension and stroke

The clinic-demographic characteristics of participants in the cohort study of hypertension and stroke are shown in Supplementary Table 3. No significant associations between selected *DIAPH1* gene variants and hypertension were observed (Table 3). Regarding stroke, rs251018 GG genotype carriers showed significantly higher incidence rate than TT/TG carriers after adjusting for age, gender, TC, TG, HDL-C, LDL-C, smoking, drinking, BMI, T2DM, and hypertension. Increased risk for stroke was also found for rs7703688 genotypes in the additive and recessive models (*P*=0.049 and *P*=0.042, respectively; Table 3).

**Table 3:** Association analyses of *DIAPH1* SNPs and hypertension and stroke in the cohort study.

**End point**	**SNP**	**Genotype**	**N**	**Person-years**	**Incidence density (/10^4^)**	***HR* (95% *CI*)**		
						**Additive model**	**Dominant model**	**Recessive model**
Hypertension	rs3805691	CC	347	5059.10	685.89	1.019 (0.896-1.166)	1.022 (0.874-1.206)	1.031 (0.734-1.447)
		CT	230	3383.53	682.72	*P*=0.744^a^	*P*=0.748^a^	*P*=0.862^a^
		TT	36	551.32	652.98			
	rs251018	TT	435	6315.13	690.41	0.964 (0.831-1.118)	0.960 (0.806-1.144)	0.937 (0.605-1.453)
		TG	157	2352.28	667.44	*P*=0.630^a^	*P*=0.650^a^	*P*=0.772^a^
		GG	21	326.53	643.13			
	rs251019	CC	306	4311.25	712.09	0.910 (0.808-1.026)	0.872 (0.744-1.022)	0.920 (0.712-1.19)
		CT	241	3724.56	647.06	*P*=0.123^a^	*P*=0.092^a^	*P*=0.525^a^
		TT	66	958.13	688.84			
	rs7703688	TT	503	7373.11	683.57	0.877 (0.728-1.056)	0.902 (0.733-1.111)	0.489 (0.218-1.095)
		TC	104	1470.03	707.47	*P*=0.166^a^	*P*=0.333^a^	*P*=0.082^a^
		CC	6	148.27	404.67			
	rs11954998	TT	446	6488.01	688.96	0.877 (0.745-1.032)	0.865 (0.723-1.035)	0.837 (0.46-1.524)
		TC	156	2335.90	667.84	*P*=0.113^a^	*P*=0.113^a^	*P*=0.561^a^
		CC	11	170.04	646.91			
Stroke	rs3805691	CC	104	11943.44	87.07	1.025 (0.796-1.319)	0.887 (0.663-1.186)	1.166 (0.655-2.075)
		CT	64	7919.26	80.82	*P*=0.851^b^	*P*=0.419^b^	*P*=0.601^b^
		TT	15	1281.25	117.07			
	rs251018	TT	133	14859.96	89.50	1.239 (0.943-1.627)	1.178 (0.858-1.615)	2.224 (1.081-4.574)
		TG	44	5689.45	77.34	*P*=0.124^b^	*P*=0.311^b^	*P*=0.030^b^
		GG	6	598.16	100.31			
	rs251019	CC	90	10196.63	88.26	1.111 (0.894-1.381)	1.103 (0.829-1.469)	1.264 (0.791-2.018)
		CT	74	8973.51	82.46	*P*=0.343^b^	*P*=0.501^b^	*P*=0.327^b^
		TT	19	1977.42	96.08			
	rs7703688	TT	149	17468.61	85.29	1.385 (1.001-1.918)	1.345 (0.936-1.935)	2.882 (1.038-8.004)
		TC	31	3451.09	89.83	*P*=0.049^b^	*P*=0.109^b^	*P*=0.042^b^
		CC	3	222.77	134.67			
	rs11954998	TT	129	15450.27	83.49	0.880 (0.661-1.171)	1.004 (0.731-1.379)	0.215 (0.03-1.541)
		TC	53	5283.27	100.32	*P*=0.631^b^	*P*=0.982^b^	*P*=0.126^b^
		CC	1	414.02	24.15			

a: Adjusted for age, gender, TC, TG, HDL-C, LDL-C, smoking, drinking, BMI and GLU.

b: Adjusted for age, gender, TC, TG, HDL-C, LDL-C, smoking, drinking, BMI, T2DM and hypertension

### Comparison of *DIAPH1* mRNA expression between IS and controls

**Figure 1:**
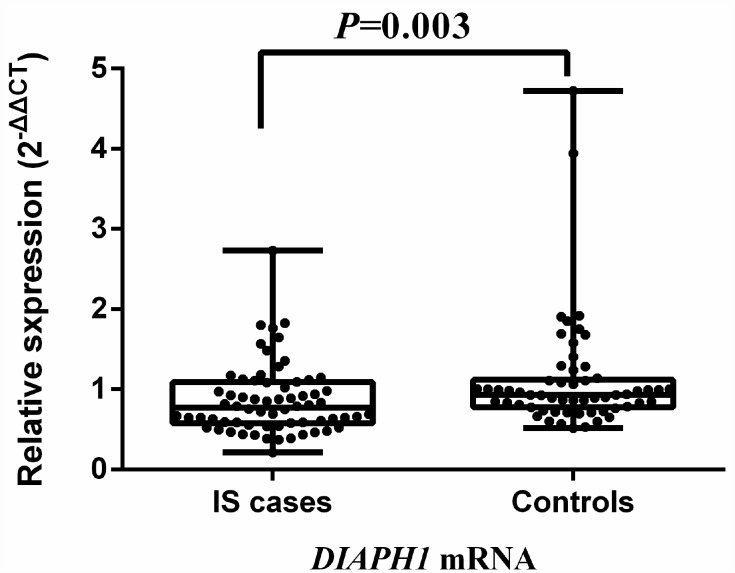
**Comparison of *DIAPH1* mRNA expression between ischemic stroke cases and controls.** The expression of *DIAPH1* mRNA (2^-∆∆CT^) in PBMCs was significantly downregulated in IS compared with controls [0.773 (0.575, 1.088) *vs* 0.933 (0.775, 1.117); *P* = 0.003]. IS, ischemic stroke.

Comparative analysis of mRNA expression for the selected SNPs was further conducted in 58 controls and 66 IS cases (43 SAO and 23 LAA). Compared with hypertensive controls, the expression of *DIAPH1* mRNA was significantly downregulated in IS [0.773 (0.575, 1.088) *vs* 0.933 (0.775, 1.117), *P =* 0.003]. Results are depicted in Figure 1. The expression of *DIAPH1* mRNA among the genotypes of rs3805691, rs251018, rs251019, rs11954998, and rs7703688 did not differ significantly, neither in IS cases nor in controls (Supplementary Figure 2). However, the *DIAPH1* mRNA level in SAO was upregulated with rs251019 genotypes, especially in homozygous CC carriers (mean expression levels in TT, TC, and CC carriers were 0.742, 0.889, and 1.765, respectively, *P_trend_* = 0.048; Supplementary Figure 3).

## DISCUSSION

The current study conducted case-control and cohort studies to investigate the associations of *DIAPH1* polymorphisms with hypertension and stroke. The key findings showed indicated that rs7703688 was significantly associated with risk of stroke, especially with IS. The significant association between rs7703688 and stroke was further validated in the cohort study. Our study also noted, for the first time, that *DIAPH1* mRNA expression is downregulated in IS, implying that *DIAPH1* might affect its pathogenesis.

Previous studies have identified an essential role for DIAPH1 in actin cytoskeletal remodeling, arterial SMC cell migration, and as a mediator of myocardial ischemia/reperfusion injury and vascular and neuroinflammatory dysfunction [16]. Whether *DIAPH1* polymorphisms affect stroke susceptibility had not been so far determined in GWAS. Rs7703688 was associated with an increased risk of IS, and modestly, but still significantly, it correlated with higher incidence of stroke in the cohort study. This suggests that rs7703688 may constitute a positive locus for stroke diagnosis and treatment. Besides, we also observed that *DIAPH1* rs3805691, rs251019 and rs11954998 were associated with IS and with two IS TOAST subtypes, i.e. SAO and LAA. However, we noticed that these three SNPs in IS cases were deviated from *HWE* (*P*<0.001), thus, the associations of the later three SNPs with IS were still need further validation.

Bioinformatics analysis for rs7703688 showed that it overlaps with a Hidden Markov Model-predicted enhancer in 15 issues, including brain, a finding that may help elucidate its involvement in stroke. The position weight matrix (PWM)-scanning process showed that the variation at rs7703688 changes the match to the AP-4 and Spz1 motifs (Supplementary Table 4). Of note, our analysis revealed that s7703688 is related to 4 eQTLs, reported by a single experiment, for *RELL2*, *ARAP3*, *FCHSD1*, and *PCDHGA6* expression. *ARAP3* encodes a phosphoinositide binding protein containing ARF-GAP and RHO-GAP, which cooperate in cell cytoskeleton remodelling and determining cell shape. Therefore, motifs coupled with eQTL data suggest that functional studies looking at whether AP-4 and Spz1 bind differentially to rs7703688 are warranted. Moreover, the GTEx Portal indicates that the rs7703688 variant is linked to lower *ARAP3* expression in whole blood. Thus, investigating whether *ARAP3* variation contributes to IS risk is also of great interest.

Accumulation of soluble forms of the receptor for advanced glycation end products (RAGEs, also known as AGER) in serum/plasma has been implicated in multiple physiological and pathological processes, including aging, diabetes, neurodegeneration, ischemia/reperfusion injury, among others [17, 18]. DIAPH1 is a key intracellular signaling effector of RAGE [19]. Mutation in the cytoplasmic domain of RAGE involving alanine substitution of R5/Q6 residues inhibits physical interaction with DIAPH1 (FH1 domain) and RAGE ligand-stimulated signal transduction. Our previous study focused on the relationship between *RAGE* genetic variations and hypertension [20]. Positive associations between *RAGE* variations and IS have been observed as well [21].

Our expression analyses of *DIAPH1* mRNA in PBMCs from IS cases and hypertensive controls showed that *DIAPH1* mRNA expression was significantly downregulated in IS. We speculate that DIAPH1 downregulation would lead to RAGEs accumulation, increasing IS risk. Besides, since DIAPH1 is also involved in the platelet release process [11], its downregulation might enhance platelet production and promote thrombosis. On the other hand, *DIAPH1* silencing improved intracellular calcium homeostasis in cardiomyocytes following I/R injury [15, 22], which suggests that DIAPH1 downregulation may be beneficial in ischemic contexts.

Since *DIAPH1* mRNA expression has an increased trend across rs251019 genotypes in SAO, especially in homozygous carriers, which might be considered as a novel eQTL for this IS subtype. Of note, a correlation between rs251019 and both *HDAC3* and *TAF7* expression has been reported [23], while another study showed that the *HDAC3* rs2530223 SNP was associated with CpG site cg24137543, which is close to the transcription start site of *DIAPH1* [24]. Although rs251019 showed a big deviation from *HWE* in our study, the significant result of rs251019 in SAO patients would still inspire us to conduct further function studies to explore its role in stroke.

The distinct advantages of the current study are reflected in the following aspects. First, the positive *DIAPH1* loci associated with stroke could be mutually validated by the case-control and cohort study design. Second, this genetic association study contains a relatively large number of IS and HS cases from south China, further classified by clinical sub-phenotype (SAO and LAA; SAH and ICH). The novel associations detected between *DIAPH1* rs7703688 and stroke may provide further insight about molecular differences between etiological stroke subtypes, especially in the Asian population.

Several limitations are also apparent in our study. First, by selecting candidate SNPs with the criterion MAF ≥ 0.05 we may have missed the chance of evaluating rare variants in *DIAPH1* also associated to stroke or hypertension. Second, all participants were from the south China Han population, so the present findings might not be representative of other demographic groups. Third, rs7703688 was not in *HWE* neither in controls within the case-control study of hypertension. Although the association of rs7703688 with stroke was further validated in the cohort study, the *P* values were not significant after multiple testing correction. Indeed, we should be cautious about the association of rs7703688 with stroke, until larger scale population studies validate these findings.

In conclusion, the current study reports original evidence for the association of genetic variation in the *DIAPH1* gene with stroke risk, especially the SAO subtype of IS. In parallel, down regulation of *DIAPH1* expression was observed in IS, suggesting that *DIAPH1* mRNA level might be a potential biomarker for IS diagnosis. Further work to elucidate the specific influence of *DIAPH1* gene variation on cerebrovascular conditions may help discover new pharmacological targets and design better therapies against stroke.

## MATERIALS AND METHODS

### Study population

Case-control and cohort studies were conducted to investigate the association of polymorphisms in the *DIAPH1* gene with hypertension and stroke. A total of 4,128 participants from the community hypertension survey were recruited between 2009 and 2010 from Guanlin and Xushe towns in Yixing city (Jiangsu, China). In the case-control study of hypertension, 2,012 patients with hypertension and 2,116 controls were recruited. Hypertension was determined as systolic blood pressure (SBP) ≥140 mmHg and/or diastolic blood pressure (DBP) ≥90 mmHg, or currently receiving anti-hypertensive medication. Subjects who had a clinical history of secondary hypertension were excluded. As the average age was higher in hypertension cases than in controls, 94 elder controls were selected from local communities for age-matching with hypertension cases. The demographic characteristics of the sample population are listed in Supplementary Table 1, and have been previously reported [25].

In the case-control study of stroke, 2,212 IS and 754 HS cases were recruited between 2013 and 2017 in the Peopleʼs Hospital of Yixing City. All stroke cases were admitted with first-time acute stroke within 72 hours. Individuals older than 85 years were excluded from the study. 2,590 age (± 5 years)- and gender-matched controls were selected from community members. The demographic and clinical characteristics of the studied population are summarized in Supplementary Table 2.

IS and HS sub-types were confirmed by a neurologist according to medical records of computed tomography (CT) and/or magnetic resonance imaging (MRI). As per TOAST criteria [26], 2,212 IS cases were classified into 1,199 SAO, 882 LAA, 108 cardiogenic cerebral embolisms (CE), 12 strokes of undetermined etiology (SUE), and 11 strokes of other determined etiology (SOE). In turn, 754 HS cases were classified into 103 SAH and 651 intracerebral hemorrhages (ICH).

For cohort studies, 2,116 participants with normal blood pressure were enrolled for the hypertension study, while 4,098 subjects were enrolled for the study assessing stroke. The clinic-demographic characteristics of these populations are listed in Supplementary Table 3. During a median follow-up time of 5.01 years, 613 cases of hypertension and 183 strokes (171 IS and 12 HS) were recorded. The flow chart of the study design is outlined in Supplementary Figure 1.

Interviews, physical examinations, and laboratory tests were conducted for all participants. Demographic characteristics including age, gender, smoking status and drinking status were obtained by trained research staff though a standard questionnaire. Weight, height, and blood pressure measurements were obtained by trained assistants according to standard protocols.

Drinking habit was defined as self-reported drinking frequency (current or past consumption of an alcoholic beverage at least 2 times per week for at least 6 months per year). Smoking habit was defined as current or past consumption of at least 20 cigarettes per week for at least 3 months per year.

The research protocol was approved by the ethics committee of Nanjing Medical University. All participants were informed in detail about the investigation and voluntarily signed the informed consent form.

### SNP selection

We selected SNPs covering the *DIAPH1* gene within 5 kb upstream and 2 kb downstream of the 5' and 3’ ends of the transcript, respectively, according to the International Hap MAP Project database (HapMap Data Rel 24/phase II Nov08, on NCBI B36 assembly, dbSNPb126). All SNPs were selected with the criteria of minor allele frequency (MAF) ≥0.05 and linkage disequilibrium (LD) r^2^ ≥0.8. SNPs with predictive biological effects and functions were obtained from HaploReg v4.1 [27] and rerun as tagSNPs. Finally, five tagSNPs, rs3805691, rs251018, rs251019, rs7703688, and rs11954998 were selected. Corresponding biological information, primers, and probes are summarized in Supplementary Tables 4, 5.

### Blood sampling and SNP genotyping

Blood samples were collected in EDTA-containing receptacles after overnight fasting (>10 h), and TC, TG, HDL-C, LDL-C, and GLU were measured. Stroke cases consented to donate 5 ml venous blood to the Department of Neurology after admission. Anticoagulated samples were stored at −20°C within 24 h after separating serum and plasma.

Genomic DNA was isolated applying a standard phenol-chloroform method. Genotyping was performed using the TaqMan allelic discrimination assay on a 7900HT Real-time PCR System (Applied Biosystems, Foster City, CA). Each plate included blank samples as negative controls to verify genotyping quality. Call rates for each SNP were > 99.9%.

### *DIAPH1* mRNA measurement

To explore whether *DIAPH1* was differentially expressed between IS cases and controls, 66 inpatients with newly diagnosed IS were recruited from Yixing People's Hospital (from Jan to Nov. 2017). Considering that hypertension is a dominant characteristic of IS, we selected 58 age- and gender- matched hypertensive controls to compare the expression of *DIAPH1* at the mRNA level. To this end, PBMCs were isolated within 4 h after blood extraction and *DIAPH1* mRNA was isolated and quantified using a standard protocol (Supplementary Methods). Primer sequences for *DIAPH1* and control GAPDH mRNAs are listed in Supplementary Table 6. In addition, DNA genotyping was conducted in both groups to investigate potential association of *DIAPH1* polymorphisms with mRNA level variation. Clinic-demographic characteristics of IS cases and hypertensive controls are summarized in Supplementary Table 7.

### Statistical analysis

Unpaired Student’s t-tests were used to assess inter-group differences for quantitative variables, presented as means ± *SD*. *HWE* for genotype frequencies was estimated with a Fisher's exact test in controls. For comparisons between not normally distributed independent samples, the Mann–Whitney U test was applied. Unconditional logistic regression was used to estimate odds ratios (*OR*s) and 95% confidence intervals (*CI*s) as well as to adjust for covariates. Cox regression was applied to estimate hazard ratios (*HRs*) and 95% *CIs* in the cohort study. A two-tailed *P* value of 0.05 was defined as the cutoff for statistical significance. All statistical analyses were performed with SPSS version 18.0 (SPSS, Inc., Chicago, IL).

## Supplementary Material

Supplementary Materials

Supplementary Figures

Supplementary Tables
