# Supplementary material for: Association of DIAPH1 gene polymorphisms with ischemic stroke
Source: Aging (Albany NY). 2020 Jan 3;12(1):416–35. doi: 10.18632/aging.102631 (PMC6977662; doi:10.18632/aging.102631)
Supplement: Supplementary Materials [file aging-12-102631-s001..pdf]

SUPPLEMENTARY MATERIALS

METHODS

Determination of *DIAPH1* mRNA expression in peripheral blood mononuclear cells

Total RNA was isolated from peripheral mononuclear cells (100μl) using the RNA Blood Kit (Cat#Yu-B02-1, Yuan Corp., Wuxi, China) according to the manual's instructions. A total of 0.3 μg total RNA was used for cDNA synthesis using TAKARA reverse transcription kits (RR047A Takara PrimeScript™ RT reagent Kit with gDNA Eraser, Japan). Primers were designed on Primer Premier 5.0 software and are shown in Supplementary Table 2. The qPCR reactions were performed in a 10 μl reaction mixture of 2 μl cDNA, 5 μl Hieff™ qPCR SYBR® GEEN Master Mix, 2.6 μl nuclease-free water, 0.2 μl upstream primer, and 0.2 μl downstream primer. The qPCR conditions were 95℃ for 5 min, 95℃ for 10 s, 60℃ for 20 s and 72℃ for 20 s (40 cycles). A melting curve cycle at 95°C for 15 s, 60°C for 1 min, and 95°C for 15 s was generated post-amplification. Three parallel samples were set up with a standard deviation <0.5, and the average value was calculated. The expression of *DIAPH1* mRNA was calculated with the $2^{-\Delta\Delta CT}$ method ($\Delta CT = CT_{\text{target gene}} - CT_{\text{house keeper gene}}$, $\Delta\Delta CT = \Delta CT - \Delta CT_{\text{control average value}}$).
