# Supplementary material for: Association of DIAPH1 gene polymorphisms with ischemic stroke
Source: Aging (Albany NY). 2020 Jan 3;12(1):416–35. doi: 10.18632/aging.102631 (PMC6977662; doi:10.18632/aging.102631)
Supplement: Supplementary Figures [file aging-12-102631-s003..pdf]

SUPPLEMENTARY FIGURES

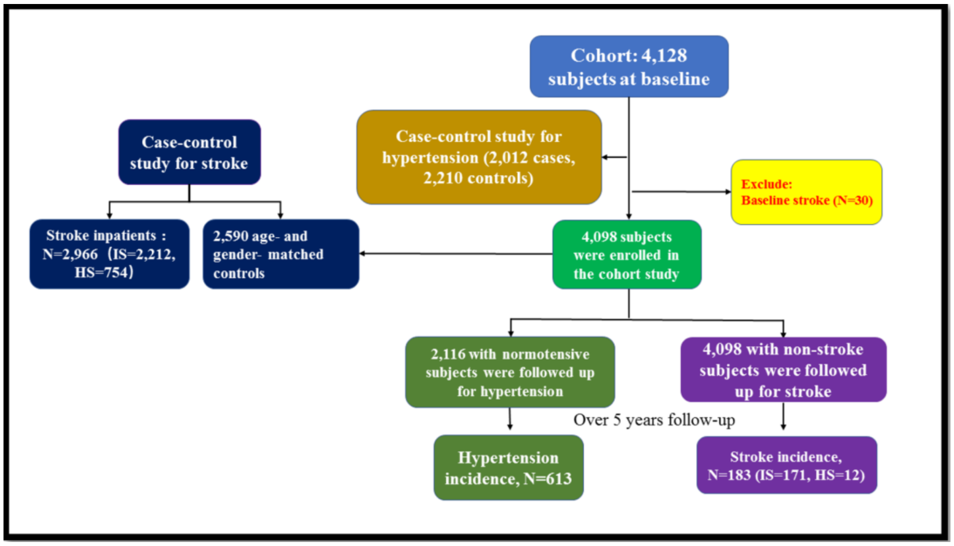

Supplementary Figure 1. Flow chart for participant selection in case-control and cohort studies evaluating associations between *DIAPH1* SNPs and hypertension and stroke. Age-matched elderly controls (n = 94) included in the hypertension case-control study, as well as hypertension cases with history of stroke (n = 30), were excluded in the cohort study of stroke.

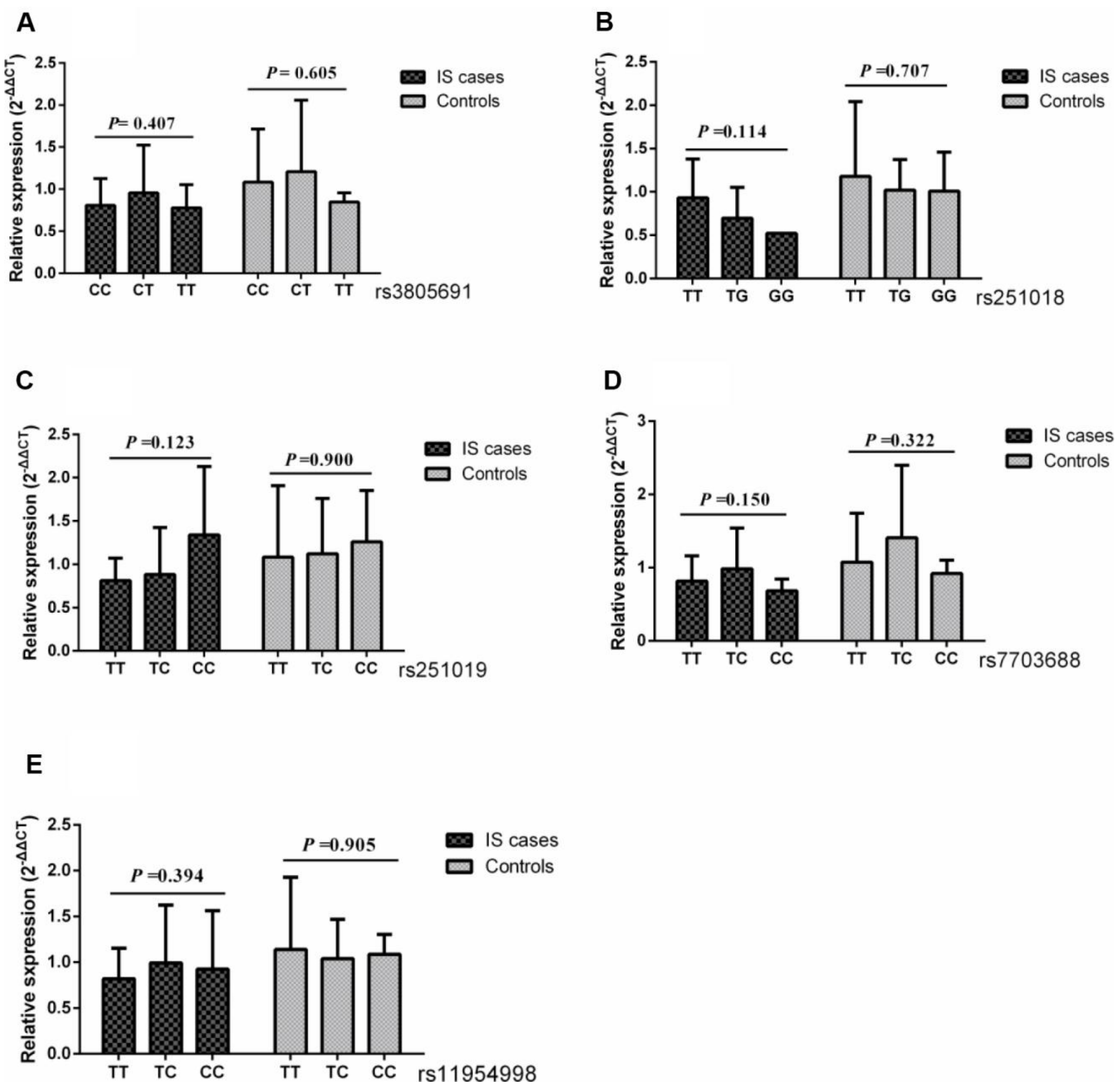

Supplementary Figure 2. Comparison of relative *DIAPH1* mRNA relative expression amongst rs3805691, rs251018, rs251019, rs11954998 and rs7703688 genotypes in IS cases and controls.

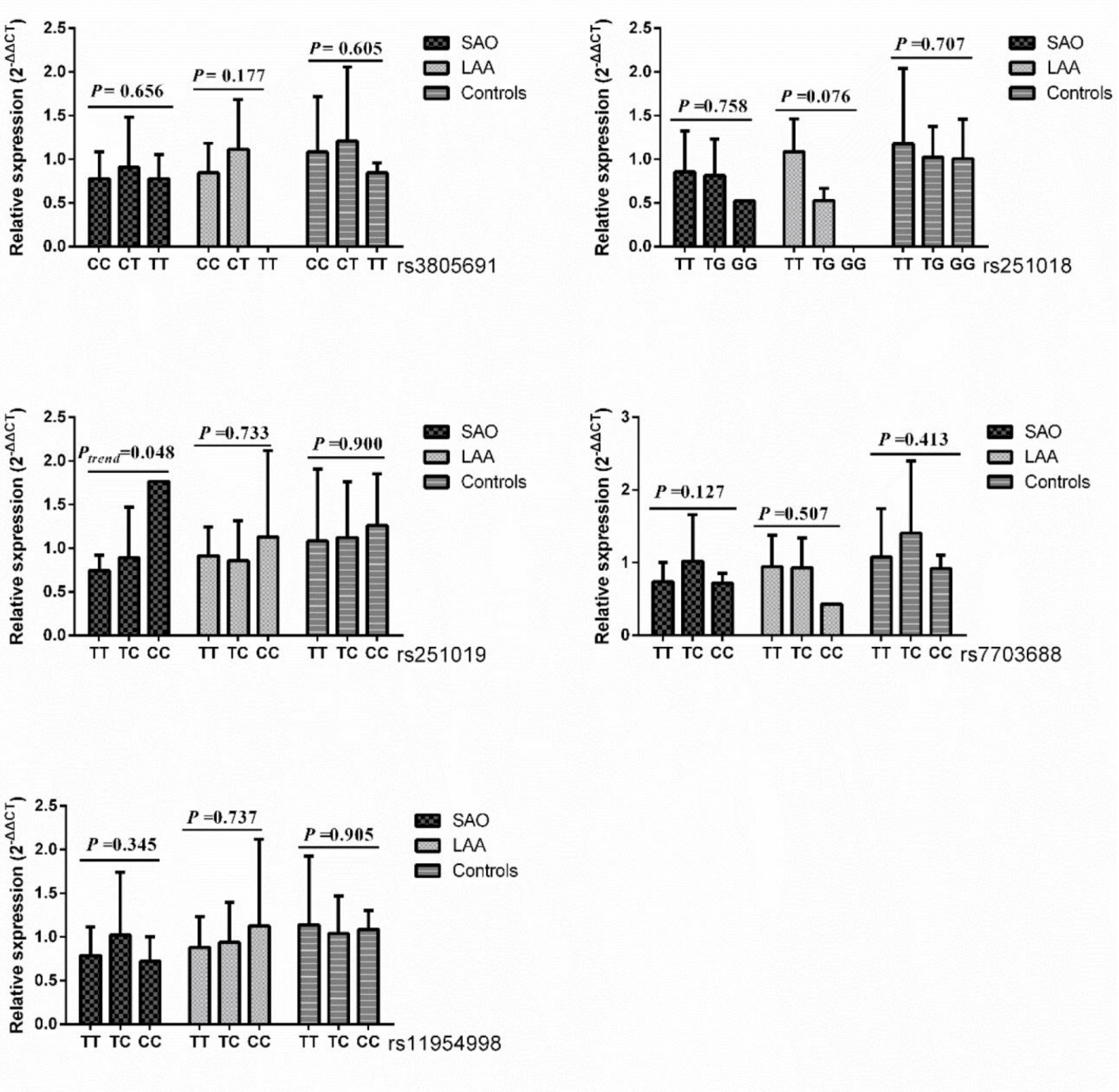

Supplementary Figure 3. Comparison of relative *DIAPH1* mRNA expression amongst rs3805691, rs251018, rs251019, rs11954998 and rs7703688 genotypes in IS subtypes and controls. *DIAPH1* mRNA level in SAO has an increased trend among rs251019 genotypes; mean expression levels for TT, TC, and CC carriers were 0.742, 0.889, and 1.765, respectively (P_{trend} = 0.048).
