# Supplementary material for: Association of DIAPH1 gene polymorphisms with ischemic stroke
Source: Aging (Albany NY). 2020 Jan 3;12(1):416–35. doi: 10.18632/aging.102631 (PMC6977662; doi:10.18632/aging.102631)
Supplement: Supplementary Tables [file aging-12-102631-s002..pdf]

SUPPLEMENTARY TABLES

Supplementary Table 1. Demographic and clinical characteristics of the case-control study of hypertension.

Characteristics	Group	Hypertension n = 2,012	Normotension n=2,210	t/χ^2	*P*
Age (year)		62.35±10.73	58.93±10.45	10.484	<0.001
Gender (%)	Male	829(41.20%)	884(40.00%)	0.632	0.427
	Female	1,183（58.80%）	1,326(60.00%)		
SBP (mm Hg)		142.86±14.30	124.24±11.36	47.018	<0.001
DBP (mm Hg)		87.53±8.54	79.08±6.51	36.369	<0.001
BMI (kg/m^2)		24.76±3.51	23.64±3.20	10.844	<0.001
TC (mmol/L)		4.94±1.05	4.78±1.01	4.574	<0.001
TG (mmol/L)		1.86±1.58	1.53±1.21	7.618	<0.001
HDL-C (mmol/L)		1.36±0.33	1.36±0.33	0.175	0.751
LDL-C (mmol/L)		2.80±0.89	2.64±0.73	6.227	<0.001
GLU (mmol/L)		5.83±2.05	5.46±1.61	6.684	<0.001
Smokers (%)	Yes	480(23.86%)	533(24.12%)	0.039	0.843
	No	1,532(76.14%)	1,677(75.88%)		
Drinkers (%)	Yes	424(21.07%)	477(21.58%)	0.163	0.686
	No	1,588(78.93%)	1,733(78.42%)		
T2DM	Yes	272 (13.52%)	196 (8.86%)	23.106	<0.001
	No	1740 (86.48%)	2014 (91.14%)		
Antihypertension medications	Compound reserpine	307 (15.26%)	-	-	-
	Zhen Ju Jiang Ya tablets	112 (5.57%)	-	-	-
	Jiang Ya tablets	221 (10.98%)	-	-	-
	Other medications	398 (19.78%)	-	-	-
	Untreated	974 (48.41%)	-	-	-

BMI, body mass index; DBP, diastolic blood pressure; GLU, glucose; HDL-C, high-density lipoprotein cholesterol; LDL-C, low-density lipoprotein cholesterol; SBP, systolic blood pressure; TC, total cholesterol; TG, triglyceride; T2DM, type 2 diabetes mellitus. Zhen Ju Jiang Ya tablets and Jiang Ya tablets natural herbal supplements used in traditional Chinese medicine.

Supplementary Table 2. Demographic and clinical characteristics of the case-control study of stroke.

Characteristics	Group	Controls n=2,590	IS n=2,212	HS n=754	F/χ^2	*P*
Age (year)		66.08±10.30	66.53±10.30	63.00±13.77**	30.931	<0.001
Gender (%)	Male	965(37.26%)	1327(59.99%)	453(60.08%)	286.404	<0.001
	Female	1,625(62.74%)	885(40.01%)	301(39.92%)		
TC (mmol/L)		0.95±0.02	1.91±0.04*	1.07±0.06*	7.287	0.001
TG (mmol/L)		1.33±0.03	1.66±0.04	1.23±0.05**	8.170	<0.001
HDL-C(mmol/L)		0.34±0.01	0.35±0.01**	0.44±0.02*	91.513	<0.001
LDL-C (mmol/L)		0.77±0.01	2.00±0.05*	0.82±0.04	5.004	0.007
Smokers (%)	Yes	573(22.12%)	531(24.00%)	124(16.44%)	17.82	<0.001
	No	2,017(77.88%)	1,681(76.00%)	630(83.56%)		
Drinkers (%)	Yes	526(20.31%)	342(15.46%)	96(12.73%)	31.776	<0.001
	No	2,064(79.69%)	1,870(85.54%)	658(87.27%)		
Hypertension (%)	Yes	1,218(47.03%)	1,536(69.44%)	486(64.46%)	263.074	<0.001
	No	1,372(52.97%)	676(30.56%)	268(35.54%)		
T2DM (%)	Yes	299(11.54%)	586(26.49%)	93(12.33%)	199.766	<0.001
	No	2,291(88.46%)	1,626(73.51%)	661(87.67%)		

HDL-C, high-density lipoprotein cholesterol; LDL-C, low-density lipoprotein cholesterol; TC, total cholesterol; TG, triglyceride; T2DM, type 2 diabetes mellitus; IS: ischemic stroke; HS: hemorrhagic stroke.
* and ** indicates that statistical differences when compared to controls.

Supplementary Table 3. Clinical and demographic characteristics of the cohort study of hypertension and stroke.

Characteristics	Group	Hypertension n=2,116	Stroke n=4,098
Age (year)		58.41±10.28	60.27±10.67
Gender (%)	Male	853(40.31%)	1660(40.51%)
	Female	1,263(59.69%)	2,438(59.49%)
BMI (kg/m^2)		23.70±3.21	22.21±3.38
TC (mmol/L)		4.78±1.01	4.85±1.03
TG (mmol/L)		1.54±1.21	1.69±1.41
HDL-C (mmol/L)		1.37±0.33	1.37±0.33
LDL-C (mmol/L)		2.65±0.72	2.72±0.81
GLU (mmol/L)		5.47±1.62	5.65±1.86
Smokers (%)	Yes	525(24.81%)	995(24.28%)
	No	1,591(75.19%)	3103(75.72%)
Drinkers (%)	Yes	468(22.12%)	883(21.55%)
	No	1,648(77.88%)	3,215(78.45%)
Hypertension (%)	Yes	-	1,985(48.43%)
	No	2,166(100%)	2,113(51.56%)
T2DM (%)	Yes	193(9.12%)	461(11.98%)
	No	1,923(90.88%)	3,637(88.02%)

BMI, body mass index; DBP, diastolic blood pressure; GLU, glucose; HDL-C, high-density lipoprotein cholesterol; LDL-C, low-density lipoprotein cholesterol; SBP, systolic blood pressure; TC, total cholesterol; TG, triglyceride, T2DM, type 2 diabetes mellitus.

Supplementary Table 4. Biological information and function prediction for selected tagSNPs.

SNP	Chr	dbSNP function	Allele	eQTL hits	Motifs changed	Promoter histone marks	Enhancer histone marks
rs3805691	5	intronic	C/T	--	Cart1, Hltf, lrf, STAT	7 tissues	14 tissues
rs251018	5	intronic	T/G	4 hits	Foxp3,Nanog,Pou5f1	STRM	16 tissues
rs251019	5	intronic	C/T	2 hits	13 altered motifs	BLD	13 tissues
rs7703688	5	intronic	T/ C	4 hits	AP-4,Spz1	--	15 tissues
rs11954998	5	intronic	T/C	--	--	--	FAT

SNP, single nucleotide polymorphism; eQTL, expression quantitative trait locus;
1000G Phase 1 population for LD calculation was set as ASN.

Supplementary Table 5. Primers and probes of selected tagSNPs in DIAPH1.

SNP		Sequence（5'-3'）	
rs3805691	probe	CCAGTAATTGAAATCATATG	CCAGTAATTGAAACCATAT
	primer	GGTAGGGAATCTGCAGCAAGAA	TTTCACTACCAGAAGCATGTCTTTG
rs251018	probe	CTGCATCAGACTTT	CCTGCATCAGAATT
	primer	TTGACCCCGTCGGCTAAGT	ATAGTTCCACAGGAAGAGGCTTAAAAG
rs251019	probe	ACAAGAAACATAATTAGGAGAGT	ACAAGAAACATAATTAGCAGAG
	primer	GCTTATTCTCCCCCCTACTTTTGTA	AGGGAAGAGGAAACTAGGACAGATG
rs7703688	probe	TGAGGGTAACATAGCTA	TGAGGGTAACACAGCTA
	primer	CAGAACACTTAATAATCTCTCCAGGAAT	GGCTTATTGTATGCTAGGTGAACTTG
rs11954998	probe	TGCAAATATGGCCTATGT	TGCAAATATGGCCCATG
	primer	TTGTGATCCGCCCACCTT	GCTTATTCTCCCCCCTACTTTTCGTA

Supplementary Table 6. Primer sequences for mRNA quantification.

Gene	Primer sequences (5'-3')	Amplification products	T_m
DIAPH1	F: GGAGTTACGATAGCCGGAACA	96bp	60℃
	R: CTTCTGTCTCCAACATGGTCTTG		
GAPDH	F: ACACACTCAGTGAACA	76bp	60℃
	R: ATGGACTCATCTTTGCCTATTGC		

Supplementary Table 7. Sample characteristics of the *DIAPH1* mRNA analysis

Characteristics	Group	IS	Controls		
		n=66	n=58	t/χ^2	P
Age (year)		65.31±6.83	64.65±7.41	0.516	0.607
Gender (%)	Male	37(56.06%)	27(46.55%)	1.118	0.290
	Female	29(43.94%)	31(53.45%)		
Smokers (%)	Yes	13(19.69%)	11(18.96%)	0.011	0.918
	No	53(80.31%)	47(81.04%)		
Drinkers (%)	Yes	21(31.82%)	18(31.03%)	5.552	0.018
	No	45(68.18%)	40(68.97%)		
Diabetes (%)	Yes	12(18.18%)	6(10.34%)	1.528	0.216
	No	54(81.82%)	52(89.66%)		
IS subtype	SAO	43(65.15%)	-	-	-
	LAA	23(34.85%)	-		

IS, ischemic stroke; LAA, large-artery atherosclerosis; SAO, small artery occlusion.

Supplementary Table 8. Association analysis of *DIAPH1* SNPs and IS subtypes in the case-control study.

TOAST subtypes	SNP	Group	WT/HT/MT	*OR* (95% *CI*)[a]		
				Additive model	Dominant model	Recessive model
SAO	rs3805691		CC/CT/TT			
		Case	794/326/56	0.758(0.663-0.866)	0.686(0.583-0.806)	0.844(0.599-1.190)
		Control	1434/990/166	$P=4.400\times10^{-5}$	$P=5.000\times10^{-6}$	$P=0.333$
	rs251018		TT/TG/GG			
		Case	821/663/22	1.070(0.920-1.243)	1.109(0.936-1.314)	0.844(0.494-1.442)
		Control	1857/663/70	$P=0.380$	$P=0.233$	$P=0.534$
	rs251019		CC/CT/TT			
		Case	714/374/110	0.817(0.724-0.922)	0.690(0.590-0.806)	1.096(0.837-1.436)
		Control	1277/1082/231	$P=0.001$	$P=3.000\times10^{-6}$	$P=0.505$
	rs7703688		TT/TC/CC			
		Case	869/296/32	1.777(1.502-2.103)	1.865(1.545-2.252)	2.810(1.534-5.149)
		Control	2164/401/25	$P=2.001\times10^{-11}$	$P=8.974\times10^{-11}$	$P=0.001$
	rs11954998		TT/TC/CC			
		Case	705/433/31	1.571(1.356-1.815)	1.675(1.421-1.984)	1.675(1.029-2.728)
		Control	1893/644/53	$P=1.760\times10^{-9}$	$P=1.139\cdot10^{-9}$	$P=0.038$
LAA	rs3805691		TT/TC/CC			
		Case	558/259/46	0.847(0.731-0.981)	0.774(0.646-0.927)	1.015(0.702-1.468)
		Control	1434/990/166	$P=2.700\times10^{-4}$	$P=0.005$	$P=0.935$
	rs251018		TT/TC/CC			
		Case	613/245/20	1.006(0.847-1.194)	1.015(0.836-1.231)	0.953(0.535-1.697)
		Control	1857/663/70	$P=0.945$	$P=0.883$	$P=0.870$
	rs251019		TT/TC/CC			
		Case	521/278/81	0.794(0.693-0.911)	0.673(0.564-0.803)	1.018(0.749-1.385)
		Control	1277/1082/231	$P=0.001$	$P=1.100\times10^{-5}$	$P=0.908$
	rs7703688		TT/TC/CC			
		Case	659/194/27	1.805(1.498-2.175)	1.831(1.483-2.262)	3.715(1.984-6.956)
		Control	2164/401/25	$P=5.710\times10^{-10}$	$P=1.955\times10^{-8}$	$P=4.100\times10^{-5}$
	rs11954998		TT/TC/CC			
		Case	562/280/24	1.446(1.225-1.708)	1.500(1.242-1.812)	1.751(1.027-3.988)
		Control	1893/644/53	$P=1.400\times10^{-6}$	$P=2.600\times10^{-5}$	$P=0.040$

SAO, small artery occlusion; LAA, large-artery atherosclerosis.
a, adjusted for age, gender, TC, TG, HDL-C, LDL-C, smoking status, drinking status, T2DM and hypertension.

Supplementary Table 9. Association analysis of *DIAPH1* SNPs and HS subtypes in the case-control study.

HS subtypes	SNP	Group	WT/HT/MT	*OR* (95% *CI*)[a]		
				Additive model	Dominant model	Recessive model
SAH	rs3805691		CC/CT/TT			
		Case	64/32/6	0.555(0.289-1.065)	0.590(0.287-1.210)	0.963(0.412-2.252)
		Control	1434/990/166	*P*=0.077	*P*=0.150	*P*=0.931
	rs251018		TT/TG/GG			
		Case	78/16/7	0.908(0.454-1.813)	0.858(0.392-1.878)	1.254(0.164-9.572)
		Control	1857/663/70	*P*=0.784	*P*=0.702	*P*=0.827
	rs251019		CC/CT/TT			
		Case	56/30/15	0.953(0.566-1.604)	0.772(0.390-1.528)	1.558(0.567-4.285)
		Control	1277/1082/231	*P*=0.855	*P*=0.457	*P*=0.390
	rs7703688		TT/TC/CC			
		Case	79/17/6	1.602(1.801-3.205)	1.362(0.592-3.134)	6.487(1.371-30.706)
		Control	2164/401/25	*P*=0.184	*P*=0.467	*P*=0.018
	rs11954998		TT/TC/CC			
		Case	71/26/6	1.066(0.550-2.069)	0.902(0.420-1.940)	3.150(0.676-14.679)
		Control	1893/644/53	*P*=0.849	*P*=0.792	*P*=0.144
ICH	rs3805691		CC/CT/TT			
		Case	347/264/35	1.019(0.826-1.258)	1.077(0.834-1.392)	0.807(0.454-1.432)
		Control	1434/990/166	*P*=0.858	*P*=0.569	*P*=0.463
	rs251018		TT/TG/GG			
		Case	470/160/17	0.982(0.763-1.264)	1.001(0.752-1.332)	0.806(0.331-1.963)
		Control	1857/663/70	*P*=0.890	*P*=0.996	*P*=0.635
	rs251019		CC/CT/TT			
		Case	336/254/52	0.926(0.800-1.070)	1.002(0.776-1.295)	1.075(0.680-1.698)
		Control	1277/1082/231	*P*=0.296	*P*=0.986	*P*=0.757
	rs7703688		TT/TC/CC			
		Case	531/109/9	0.958(0.695-1.319)	0.952(0.672-1.350)	0.962(0.255-3.626)
		Control	2164/401/25	*P*=0.792	*P*=0.783	*P*=0.954
	rs11954998		TT/TC/CC			
		Case	476/155/14	1.109(0.859-1.433)	1.140(0.857-1.516)	0.940(0.349-2.532)
		Control	1893/644/53	*P*=0.429	*P*=0.365	*P*=0.902

SAH, subarachnoid hemorrhage; ICH, intracerebral hemorrhage.
a: adjusted for age, gender, TC, TG, HDL-C, LDL-C, smoking status, drinking status, T2DM and hypertension.
